# Non-steroidal Anti-inflammatory Drugs (NSAIDs) Systemic Use: The Risk of Renal Failure

**DOI:** 10.3389/fped.2019.00517

**Published:** 2020-01-24

**Authors:** Albert Bensman

**Affiliations:** Service de Nephrologie Pediatrique, Hopital Necker–Enfants Malades, Paris, France

**Keywords:** non-steroidal anti-infammatory drugs, volume depletion, acute renal failure, arachidonic acid, prostaglandin E2, interstitial nephritis

## Abstract

For the pediatric nephrologist, the over-the-counter status for non-steroidal anti-inflammatory drugs (NSAIDs) is surprising due to their possible harmful side effects. These can include acute renal failure due mainly to glomerular hypoperfusion which may lead to acute tubular necrosis; more rarely in children, medullary ischemic injury and cardiovascular diseases; acute or chronic interstitial nephritis which may cause chronic renal failure. All of them may create electrolyte abnormalities: hyponatremia, hyperkaliemia, renal tubular acidosis, fluid retention causing hypertension.

Non-steroidal anti-inflammatory drugs (NSAIDs) are over-the-counter drugs largely used in the pediatric population, including in very young children (1). For the pediatric nephrologist, this over-the-counter status is surprising due to their possible harmful side effects, in relation with different mechanisms.

## The Role of Prostaglandins E2 (PGE2) in the Kidney

Several studies have shown the vasodilatory effects of arachidonic acid in the kidney. The renal microvasculature displays segmental heterogeneity but PGE2 has an important role in the preglomerular microvessels (2). In case of volume depletion, such as dehydration and acute bleeding, but also in cases of nephron reduction, there is a decrease in the preglomerular microvessels blood circulation. The prostaglandins, mainly PGE2, by keeping a preglomerular vasodilatation are able to maintain a normal or a satisfactory glomerular filtration rate (GFR).

NSAIDs inhibit the prostaglandin secretion in the kidney. In case of volume depletion, it is easy to understand the renal dangers of prescribing NSAIDs which block the physiological mechanism for keeping the GFR in the best conditions.

## Acute Renal Failure and Hypoperfusion

We published our results of acute renal failure (3). Within 20 months, seven children presenting with diarrhea and/or vomiting and fever were treated with therapeutic doses (range 11.5–32 mg/kg per day) of ibuprofen for 1–3 days before developing acute renal failure. The range of maximum plasma creatinine levels was 180–650 micromol/l. One patient required emergency dialysis for hyperkalaemia, uraemia, and hyperphosphataemia. Between 3 and 9 days after cessation of NSAID and adequate treatment including rehydration, all patients recovered completely with normalized creatinine levels. Once the acute phase was controlled, the long term outcome was excellent.

A much wider study by Misurac et al. (4) allowed the conclusion that non-steroidal anti-inflammatory drugs are an important cause of acute kidney injury (AKI) in children (4); 1015 children with AKI were identified. Twenty-one children had clinical, laboratory, and radiographic studies suggesting NSAID associated acute tubular necrosis. Fifteen of 20 children (75%) for whom data were available received NSAIDs within recommended dosing limits. Patients under 5 years old were more likely to require dialysis, intensive care unit admission, and a longer length of stay (median 10 vs. 7 days if older). The fact that young children with NSAID associated AKI may have increased disease severity lead to the conclusion that their dosage should be reduced as often as possible.

NSAID related nephrotoxicity remains an important clinical problem in the newborns (5) in whom the functionally immature kidney may exert a significant effect on the disposition of the drugs. For the same reasons, a child with a decreased GFR should most of the time not receive NSAIDs. The same precaution should be taken in case of a single kidney even if the GFR is normal.

The results of the study by Misurac et al. (4) were confirmed by a prospective study over a 1-year period where 105 children with acute gastroenteritis were studied (6). Balestracci et al. reported in this study that renal impairment was more frequent in children who received ibuprofen. Ibuprofen exposure remained an independent risk factor for AKI after adjusting for the degree of dehydration. This magnitude of dehydration was the second independent risk factor of AKI after ibuprofen exposure. These findings are very interesting since it is well-known that dehydration can induce AKI by itself due to renal hypoperfusion.

## Tubulointerstitial Nephritis (TIN)

Medications rather than infection are now the leading cause of acute TIN in children (7). NSAIDs are able to cause acute TIN in children. Systemic manifestations of hypersensitivity, such as fever, rash, and eosinophilia are rare in case of NSAID induced acute TIN. NSAID induced TIN may be associated with minimal change to nephrotic syndrome (8). In the study by Misurac et al. (4), among the 27 children with AKI, 6 had findings suggesting NSAID associated acute interstitial nephritis.

A long term prescription of NSAIDs may lead to chronic interstitial nephritis, chronic renal failure (9) and cardiovascular diseases (10). These cases are described in adults, not in children, but they should be known by the pediatricians.

## Conclusion

NSAIDs are freely available over-the-counter in the pediatric population. It may lead to the conclusion that they are not dangerous, but benign drugs. In our experience and by studying the literature, we conclude that, from a nephrological point of view, they are not benign and can be dangerous. Thus, the over-the-counter status of NSAID in the pediatric population should be revised by national Drug Agencies.

## Author Contributions

The author confirms being the sole contributor of this work and has approved it for publication.

### Conflict of Interest

The author declares that the research was conducted in the absence of any commercial or financial relationships that could be construed as a potential conflict of interest. The reviewer MD declared a shared affiliation, with no collaboration, with one of the authors, AB, to the handling editor at time of review.
